# Gut fungi differentially response to the antipyretic (heat-clearing) and diaphoretic (exterior-releasing) traditional Chinese medicines in *Coptis chinensis*-conditioned gut microbiota

**DOI:** 10.3389/fphar.2022.1032919

**Published:** 2022-11-18

**Authors:** Yanan Yang, Weiying Lu, Xiaopo Zhang, Chongming Wu

**Affiliations:** ^1^ Pharmacology and Toxicology Research Center, Institute of Medicinal Plant Development, Chinese Academy of Medical Sciences & Peking Union Medical College, Beijing, China; ^2^ Reproductive Medical Center, Hainan Woman and Children’s Medical Center, Haikou, China; ^3^ Key Laboratory of Tropical Translational Medicine of Ministry of Education, Hainan Key Laboratory for Research and Development of Tropical TCMs, School of Pharmacy, Hainan Medical University, Haikou, China; ^4^ School of Chinese Materia Medica, Tianjin University of Traditional Chinese Medicine, Tianjin, China

**Keywords:** gut fungi, gut bacteria, traditional Chinese medicine (TCM), antipyretic (heat-clearing) drugs, diaphoretic (exterior-releasing) drugs, gut microbiome

## Abstract

Antipyretic (heat-clearing) and diaphoretic (exterior-releasing) drugs are two main groups of traditional Chinese medicines (TCMs) possessing anti-microbes and anti-inflammation effects, with the former mainly through clearing pyrogens while the latter through promoting diaphoresis. Although anti-microorganism is a common action of these two kinds of TCMs, their difference in antimicrobial spectrums and their interactions when combinedly used remain unclear. Herein, we prepared aqueous extracts from *Coptis chinensis* (HL) and other antipyretic or diaphoretic TCMs, orally administrated them to C57BL/6 mice at a clinical dose for fourteen days, and analyzed their impaction on both gut bacteria and fungi using full-length 16 S rRNA gene sequencing and internal transcribed spacer 1/2 (ITS1/2) gene sequencing, respectively. Oral administration of HL significantly changed the structure of gut bacteria but showed little influence on gut fungi. Co-treatment with antipyretic or diaphoretic TCMs alleviated the impact of HL on gut bacteria to a similar degree. However, combined with either heat-clearing or exterior-releasing TCMs significantly strengthened the influence of HL on gut fungi, with the latter superior to the former. The antipyretic TCMs enriched *Penicillium* spp. while diaphoretic TCMs promoted *Fusarium* spp. Further analysis revealed that the diaphoretic TCMs-enriched fungi *Fusarium* spp. were positively related to *Akkermansia* spp., a beneficial bacterium that interacts with Toll-like receptor 4 (TLR4) and regulates thermogenesis, thus providing a potential linkage with their pro-diaphoresis effect. Together, our results reveal that gut fungi differentially respond to the impact of heat-clearing and exterior-releasing TCMs on *Coptis chinensis*-conditioned gut microbiota, which provides insights into their functional characteristics.

## Introduction

Traditional Chinese medicine (TCM) has successfully protected Chinese people against numerous of epidemics for thousands of years and formed a unique theory that guides TCM physicians to treat various diseases using proper medicines (Qiao et al., 2022). Antipyretic (heat-clearing) and diaphoretic (exterior-releasing) drugs are two main groups of TCM drugs, which are widely used to treat various infectious and inflammatory diseases (Jiang and Xu, 2015; Hao et al., 2021). The antipyretic TCMs, such as *Coptis chinensis*, *Phellodendron chinense* C.K.Schneid. and Gypsum Fibrosum, are usually used to remove pyretic toxicity. For example, *Phellodendron chinense* C.K.Schneid. and Gypsum Fibrosum can significantly reverse heat stress-induced inhibition of lymphocytic proliferation (Zhu et al., 2011). Moreover, *Phellodendron chinense* C.K.Schneid. is also traditionally used for quenching fire, counteracting toxicity to ameliorate heat accumulation in the intestine and stomach (Kim et al., 2017). In contrast, diaphoretic TCMs, such as *Asarum sieboldii* Miq., *Angelica dahurica* (Hoffm.) Benth. & Hook.f. ex Franch. & Sav. and *Notopterygium incisum* K.C.Ting ex H.T.Chang, are widely used to clear heat *via* promoting sweat. *Angelica dahurica* (Hoffm.) Benth. & Hook.f. ex Franch. & Sav. possesses an anti-inflammatory effect (Wang et al., 2016) and *Notopterygium incisum* K.C.Ting ex H.T.Chang displays potential anti-fungal activity (Xiao et al., 2018). Although both two groups of TCMs can remove internal toxins and exhibit anti-microbes and anti-inflammation activities, the antipyretic drugs are mainly through removing pathogens and pyrogens, and improving immunity (Chen et al., 2018; Priya et al., 2022), while diaphoretic drugs *via* promoting diaphoresis and enhancing superficial blood circulation (Huang et al., 2009). Anti-microorganism is a common action of antipyretic and diaphoretic drugs. However, their difference in the anti-microbial spectrum and whether they are synergistic on gut microbiome when combinedly used remain largely unknown.

The bridging role of gut microbiota in maintaining human health and mediating the pharmacological effects of various drugs has cast new light on both basic medicine and clinical practice. A mounting number of studies proved that the *in vivo* efficacy of many drugs, especially TCM drugs, is largely attributed to the modulation of gut microbiota (Wu et al., 2019; Zhang et al., 2020; Dong et al., 2021; Wu C. et al., 2022; Wang et al., 2022). For example, the lipid-lowering effects of *Coptis chinensis* and its active component berberine are largely dependent on the modulation of gut microbiota (Ji et al., 2021; Wu C. et al., 2022). The anti-obesity of *Ganoderma lucidum* is transmissible *via* horizontal faces transfer from *Ganoderma lucidum*-treated mice (Chang et al., 2015). Our preliminary study also found that although both hot-natured and cold-natured herbs possess verified antidiarrheal effects, their modulation on gut microbiota is distinct, that is, cold-natured TCMs notably change the structure of gut microbiota while hot-natured TCMs exert little effect on gut microbiota (Zhang et al., 2021). Simultaneously, the gut microbial functional sets (CAGs) could act as a possible indicator to reflect TCM flavor (Yang et al., 2022). These results suggest that the gut microbiota is a crucial medium to mediate the pharmacological effects of various TCM drugs and provides a new breakthrough to interpret the traditional theories of TCM.

The gut microbiome is an extremely complex ecosystem consisting of bacteria, fungi and viruses, and archaea (Mahnic and Rupnik, 2018). Besides bacteria, over 3.8 million fungi with intricate genetic information and abundant active metabolites inhabit the intestines and participate in the regulation of human health (David L and Robert, 2017; Huseyin et al., 2017). Hitherto, many antibiotics (Wiese and Imhoff, 2019), anti-diabetes drugs (Wang et al., 2021), and anti-cancer drugs (Chu et al., 2021) used in the clinic are derived from fungi. Hence, gut fungi may play an important role in the *in vivo* efficacy of various drugs. However, current studies on gut microbiota-drug interaction are mainly focused on bacteria, and the modulation of gut fungi by drugs has been largely overlooked. To comprehensively interpret the impact of various drugs on gut microbiome and explore their pharmacological mechanisms, we should pay more attention to the response of the gut fungal community.

This is a part of a systemic investigation aiming to explore the interactions among various traditional Chinese medicines (TCMs) from the aspect of modulating gut microbiome. In this study, we mainly focused on the impact of heat-clearing/exterior-releasing TCMs on gut microbiota when combined with *Coptis chinensis*. The gut microbiota was analyzed by full-length 16 S rRNA gene sequencing and ITS1/2 gene sequencing, respectively, to monitor both gut bacterial and fungal communities. The correlation between gut bacteria and gut fungi was also analyzed. This work displayed the modulation of *Coptis chinensis* on both gut bacteria and gut fungi, and revealed the differential impacts of heat-clearing and exterior-releasing TCMs on *Coptis chinensis*-conditioned gut bacterial and fungal communities.

## Materials and methods

### Preparation of TCM pair extract

Six TCMs, named *Coptis chinensis* Franch (Huanglian, HL), *Phellodendron chinense* C.K.Schneid. (Huangbo, HB), Gypsum Fibrosum (Shigao, SG), *Asarum sieboldii* Miq. (Xixin, XX), *Angelica dahurica* (Hoffm.) Benth. & Hook.f. ex Franch. & Sav. (Baizhi, BZ) and *Notopterygium incisum* K.C.Ting ex H.T.Chang (Qianghuo, QH), were purchased from Bohaotang Chinese traditional medicinal crops, Bozhou China. All of them were authenticated by professor Nian kai Zeng and deposited in Hainan Medical University with the designations FHMU6962, FHMU6963, FHMU6971, FHMU6966, FHMU6967, FHMU6969, respectively. First, we prepared 100 g of unprocessed HL as the basic drug. Then, the other four TCMs were individually mixed with HL in a ratio of 1:1 (100 g:100 g). Afterwards, the mixed raw medicinal materials were extracted with 2000 ml distilled water twice for 120 min each time. Finally, the water extracts were merged, filtered, and concentrated to obtain the extracts of herb pairs.

### UPLC-QTOF/MS analysis of the TCM pair extracts

The ultra-high-performance liquid chromatography-high resolution time-of-flight mass spectrometry (UPLC-QTOF/MS) analysis was performed on an Agilent 1,290 Infinity II UHPLC system coupled to an Abscix Triple TOF/MS instrument. Chromatographic separation was achieved using an Inertsil ODS-3 (2.1 mm × 50 mm, 2.0 μm) analytical column operated at 40°C. The mobile phase consisted of water (A) and methanol (B) at a flow of 0.40 mlmin^− 1^. The gradient started from 90% A in 1.0 min, followed by 5% A to 95% A in 10  min, and held 95% A for 3  min, then returned to the initial gradient composition and allowed to equilibrate for 2 min. Mass spectrometric analyses were collected in positive mode with full scan mode from 100 to 1,000 m/*z*. The spray voltage was 5.5  kV, and the desolvation temperature was 550°C. The second-order mass spectrometry bombardment energy was 35 eV. The pressures of the inner coaxial nebulizer N_2_ gas (GS1), dry N2 gas (GS2), and curtain N_2_ gas (CUR) were 50, 50, and 35 psi, respectively.

### Animal experiment

This animal experiment was approved by the research animal ethics committee of the Institute of Medicinal Plant Development affiliated with the Chinese Academy of Medical Sciences (No.SLXD-20210831013, Beijing, China). All procedures were performed in accordance with the National Institutes of Health guide.

A total of 55 male C57BL/6 mice (6 weeks old, 18–20 g) were obtained from SPF (Beijing) Biotechnology Co., Ltd. (Beijing, China) and raised in a semi-barrier system with a 12 h dark-light cycle (from 8 am to 8 pm) and controlled room temperature (23 ± 2 °C) and humidity (40 ± 5%). The animals were fed on standard rat chow with free access to tap water during the experiment. After seven days of acclimatization, nine mice were randomly stratified into the HL group and orally administrated with aqueous extracts of HL. Fourteen mice were divided into QR (heat-clearing) supergroups, among which half of them were orally gavaged with HL + HB aqueous extracts and half with HL + SG aqueous extracts. Twenty-four mice were stratified into JB (exterior-releasing) supergroup, among which eight mice were given HL + XX aqueous extracts, another eight mice were given HL + BZ aqueous extracts and the remaining eight mice were given HL + QH aqueous extracts. According to the clinical dosages in Pharmacopoeia of the People’s Republic of China (2020 edition), *Coptis chinensis* Franch (Huanglian, HL), *Phellodendron chinense* C.K.Schneid. (Huangbo, HB), *Asarum sieboldii* Miq. (Xixin, XX), *Angelica dahurica* (Hoffm.) Benth. & Hook.f. ex Franch. & Sav. (Baizhi, BZ) and *Notopterygium incisum* K.C.Ting ex H.T.Chang (Qianghuo, QH) are about 5–15g/day for adults, Gypsum Fibrosum (Shigao, SG) is about 30–60 g/day. In practice, TCM physicians usually increase the dosage of the herbs based on the conditions of patients. We took 15 g/day for each herb as an adult dose in our test. So the dose for each herbal pair is 30 g/day for adults, which was corresponding to about 6 g/kg for mouse. To strengthen the modulatory effect, we took 10 g/day as the testing dose for each herbal pair in our experiment. After 14 days, fresh fecal samples were collected from each mouse and kept at −80°C for further gut microbiome analysis.

### DNA extraction and gene sequencing

The fecal bacteria DNA was extracted using FastDNA Spin Kit (MP Biomedicals, Santa Ana, United States) according to the manufacturer’s instructions. The ITS2 region of fungi and V1-V9 region of bacterial 16 S rRNA gene were amplified using TransGen AP221-02 and TransStart Fastpfu DNA Polymerase. The PacBio libraries were built and sequenced using a SEQUEL IIe system by Shanghai Biozeron Biothchnology Co., Ltd. (Shanghai, China). The sequencing data were subjected to bioinformatics data analysis including quality control, assembly, and abundance quantification.

### Bioinformatics analysis and statistics

Full-length 16 S rRNA and ITS1/2 sequencing data were subjected to analysis with R package of R version 4.0.2. Alpha- and beta-diversity of gut bacteria or gut fungi were calculated by vegan package (v2.7). The difference in Bray-Curtis distance of the PCoA, principal coordinate analysis and clustering analysis at the OTU level was assessed by Adonis analysis. The online data analysis platform MicrobiomeAnalyst was used to perform SparCC analysis (https://www.microbiomeanalyst.ca/). Correlation analysis was calculated by spearman algorithm. The interaction network of gut fungi-bacteria was visualized using Cytoscape 3.8.2 software. The differences in gut bacterial and gut fungal composition among groups were analyzed by Student’s t-test and *p* value was adjusted by FDR, and FDR< 0.05 was considered as statistical significance. GraphPad Prism 8.0 Software (San Diego, CA, United States) and ggplot2 package were used for visualization.

## Results

### Animal study design

**FIGURE 1:**
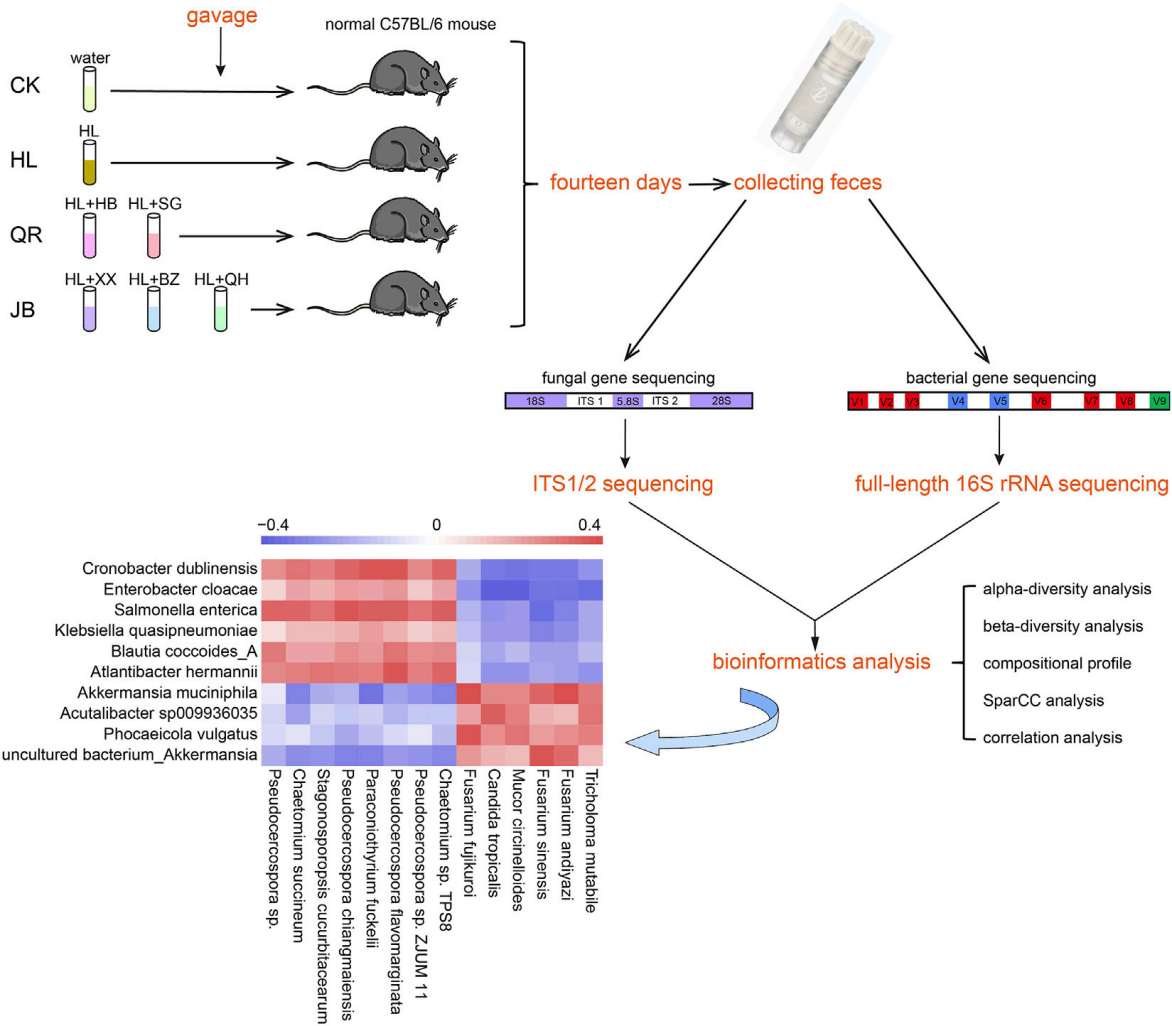
The design of experiment. Two antipyretic drugs and three diaphoretic drugs were selected to pair with *Coptis chinensis* for this study. The aqueous extract of each herbal pair was prepared and then orally administrated to male C57BL/6 mice by gavage at a clinical equivalent dose for fourteen days. The fecal DNA was extracted to conduct full-length 16 S rRNA sequencing and internal transcribed spacer 1/2 (ITS1/2) sequencing. The bioinformatics analysis was applied to investigate the alteration of gut bacterial and gut fungal community, as well as the correlation between gut bacteria and gut fungi.

To investigate the co-regulation of heat-clearing and exterior-releasing TCMs on gut microbiota, we chose *Coptis chinensis* (Huanglian, HL), a common antipyretic TCM, as the basic drug and coupled HL with another antipyretic or diaphoretic TCM with equal weight proportion. Five TCMs, namely *Phellodendron chinense* (Huangbo, HB), *Gypsum Fibrosum* (Shigao, SG), *Asari radix et rhizoma* (Xixin, XX), *Angelicae dahuricae radix* (Baizhi, BZ) and *Notopterygii rhizoma et radix* (Qianghuo, QH), were selected to pair with HL (HL + HB, HL + SG, HL + XX, HL + BZ, and HL + QH). According to the functional classification by Traditional Chinese Medicine, *Coptis chinensis*, *Phellodendron chinense* and *Gypsum Fibrosum* belong to antipyretic (heat-clearing) drugs, while *Asari radix et rhizoma*, *Angelicae dahuricae radix* and *Notopterygii rhizoma et radix* are diaphoretic (exterior-releasing) drugs. Therefore, HL + HB and HL + SG groups were further classified as heat-clearing (QR) supergroup while HL + XX, HL + BZ and HL + QH groups were termed exterior-releasing (JB) supergroup (Figure 1).

The aqueous extract of each drug pair was prepared and orally administrated to male C57BL/6 mice at a clinical equivalent dose for fourteen days. Afterwards we collected fresh fecal samples from each mouse and conducted full-length 16 S rRNA gene sequencing and internal transcribed spacer 1/2 (ITS1/2) gene sequencing. The composition of gut bacterial and fungal communities was analyzed from phylum to species levels. The SparCC analysis was used to identify co-abundance gene groups (CAGs) as functional sets of gut fungi. The relationship between gut fungi and microbiota was also assessed by spearman correlation analysis (Figure 1). In this work, we mainly focused on the co-regulatory effects of various drug pairs on gut microbiota, so we did not check the modulation of individual antipyretic or diaphoretic TCM on gut microbiome.

### Heat-clearing and exterior-releasing TCM drugs alter coptis chinensis-conditioned gut bacterial composition in a similar degree

**FIGURE 2:**
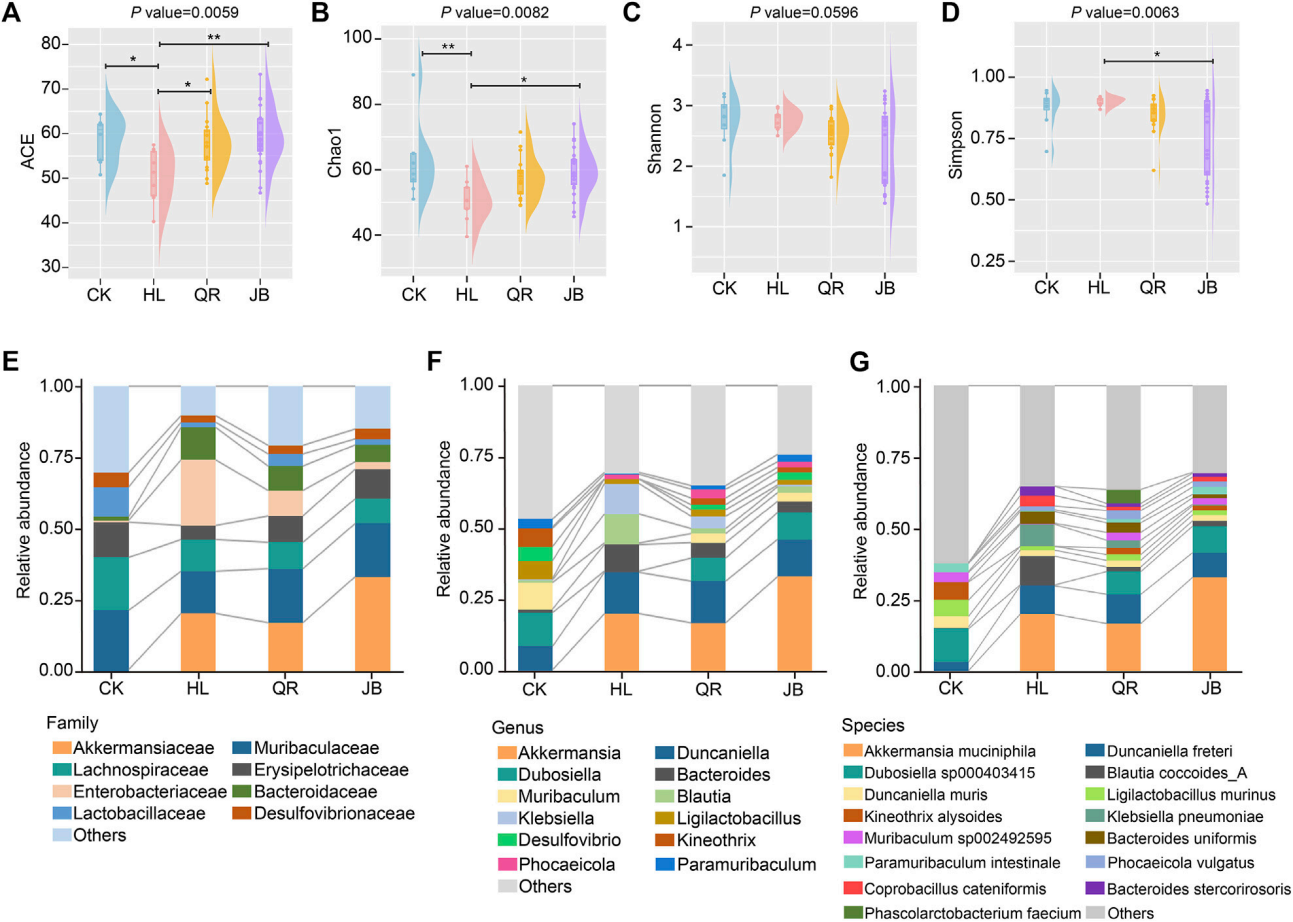
The alteration of gut microbial composition after antipyretic and diaphoretic drugs treatment. The alpha diversity of gut bacteria among four groups assessed by **(A)** ACE **(B)** chao1, **(C)** shannon and **(D)** simpson indices. The gut microbial profile diagram at **(E)** family level **(F)** genus level and **(G)** species level. *p < 0.05, **p < 0.01.

We first assessed the impaction of different heat-clearing and exterior-releasing drugs on *Coptis chinensis*-conditioned gut bacterial community by full-length 16 S rRNA gene sequencing. Four indices, namely ACE, Chao1, Shannon, and Simpson, were used to evaluate the alpha diversity of gut microbiota, in which ACE and Chao1 mainly represent the taxa richness of gut bacterial community while Shannon and Simpson reflect both richness and evenness of gut microbiota. Treated with *Coptis chinensis* remarkably decreased ACE and Chao1 but showed little effect on Shannon and Simpson (Figures 2A–D), suggesting that the antibacterial effect of *Coptis chinensis* has a broad spectrum but shows less specificity. Co-treatment with both heat-clearing and exterior-releasing drugs significantly restored ACE and Chao1 without marked change in Shannon and Simpson indices (Figures 2A–D), indicating that these TCMs can attenuate the antibacterial effect of *Coptis chinensis*.

Detailed analysis of gut bacterial composition at the family, genus, and species levels also displayed the interactions between each antipyretic/diaphoretic TCM and *Coptis chinensis* on gut bacterial richness. Eight families, *i.e.* Akkermansiaceae, Muribaculaceae, Lachnospiraceae, Erysipelotrichaceae, Enterobacteriaceae, Bacteroidaceae, Lactobacillaceae, and Desulfovibrionaceae, accounted for the major proportion of the gut bacterial community. Treatment with *Coptis chinensis* dramatically decreased short-chain fatty acids (SCFAs)-producing Erysipelotrichaceae and Lactobacillaceae while increasing opportunistic pathogen Enterobacteriaceae. Both heat-clearing and exterior-releasing TCMs essentially reversed these changes (Figure 2E). Erysipelotrichaceae and Lactobacillaceae are among the largest families in gut microbiota and both contain a large number of species. The decrease of these two families by *Coptis chinensis* will dramatically reduce the taxa richness. The bar plots in Figures 2F, G also showed an obviously decreased number of genera and species in the *Coptis chinensis*-treated group, which was restored when co-treated with heat-clearing and exterior-releasing TCMs. Especially, *Dubosiella*, *Muribaculum*, *Desulfovibrio*, *Kineothrix,* and *Paramuribaculum* were largely reduced by *Coptis chinensis* but restored by heat-clearing and exterior-releasing TCMs (Figure 2F). At the species level, *Dubosiella sp000403415*, *Kineothrix alysoides*, *Muribaculum sp002492595,* and *Paramuribaculum intestinale* were reduced while *Klebsiella pneumoniae*, *Bacteroides uniformis*, *Bacteroides stercorirosoris* and *Coprobacillus cateniformis* were increased by *Coptis chinensis*. Both antipyretic and diaphoretic TCMs reversed these changes in a similar trend (Figure 2G). Contrastively, the beneficial gut bacteria, *Akkermansia muciniphila*, was significantly increased by *Coptis chinensis* but co-treatment with heat-clearing and exterior-releasing TCMs persevered this change (Figure 2G).

### Heat-clearing and exterior-releasing TCM drugs shift the gut microbial structure in a similar way

**FIGURE 3:**
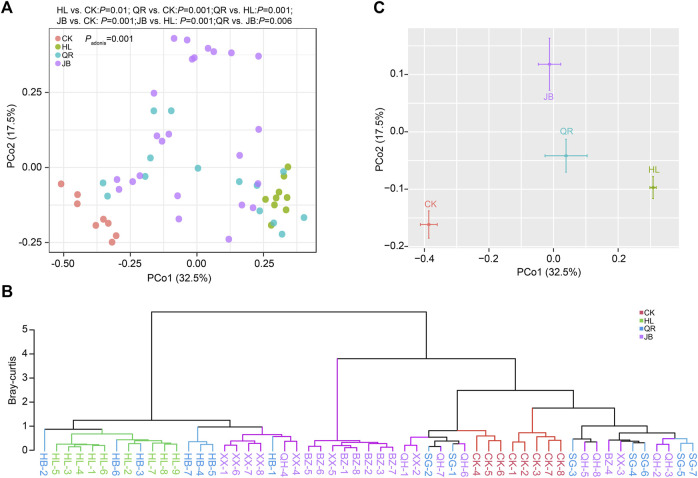
The alteration of gut microbial structure after antipyretic and diaphoretic drugs treatment. The structure of gut bacteria was assessed by Principal coordinate analysis (PCoA) which was presented by samples **(A)** or merged groups **(C)**. **(B)** Clustering analysis based on bray-curtis distance.

We then assessed the effect of different TCM combinations on the structure of gut microbiota. Principal coordinate analysis (PCoA) based on Bray-Curtis distance demonstrated that *Coptis chinensis* (HL) group was obviously separated from the normal control (CK), while combination with heat-clearing or exterior-releasing TCMs shifted the gut bacterial structure from HL status to the CK condition in a different degree (Figure 3A). Co-treatment with *Gypsum Fibrosum* (Shigao, SG) largely migrated the microbial structure towards normal, but *Phellodendron chinense* (Huangbo, HB) had little effect. Comparatively, all exterior-releasing drugs could restore the structure of gut microbiota with different intensities (Figure 3A). Cluster analysis based on Bray-Curtis distance yielded similar results. The samples in HL and CK groups were clustered and separated. The HL + HB group was clustered together with HL, the HL + XX and HL + BZ groups were clustered in the middle area, while the HL + SG and HL + QH groups were close to the control group (Figure 3B). Generally, a combination of both heat-clearing and exterior-releasing drugs could migrate the gut microbial structure from the *Coptis chinensis*-condition to normal status, and there is no obvious difference between the two types of drugs (Figure 3C).

### Heat-clearing and exterior-releasing TCMs differentially alters the coptis chinensis-conditioned gut fungal composition

**FIGURE 4:**
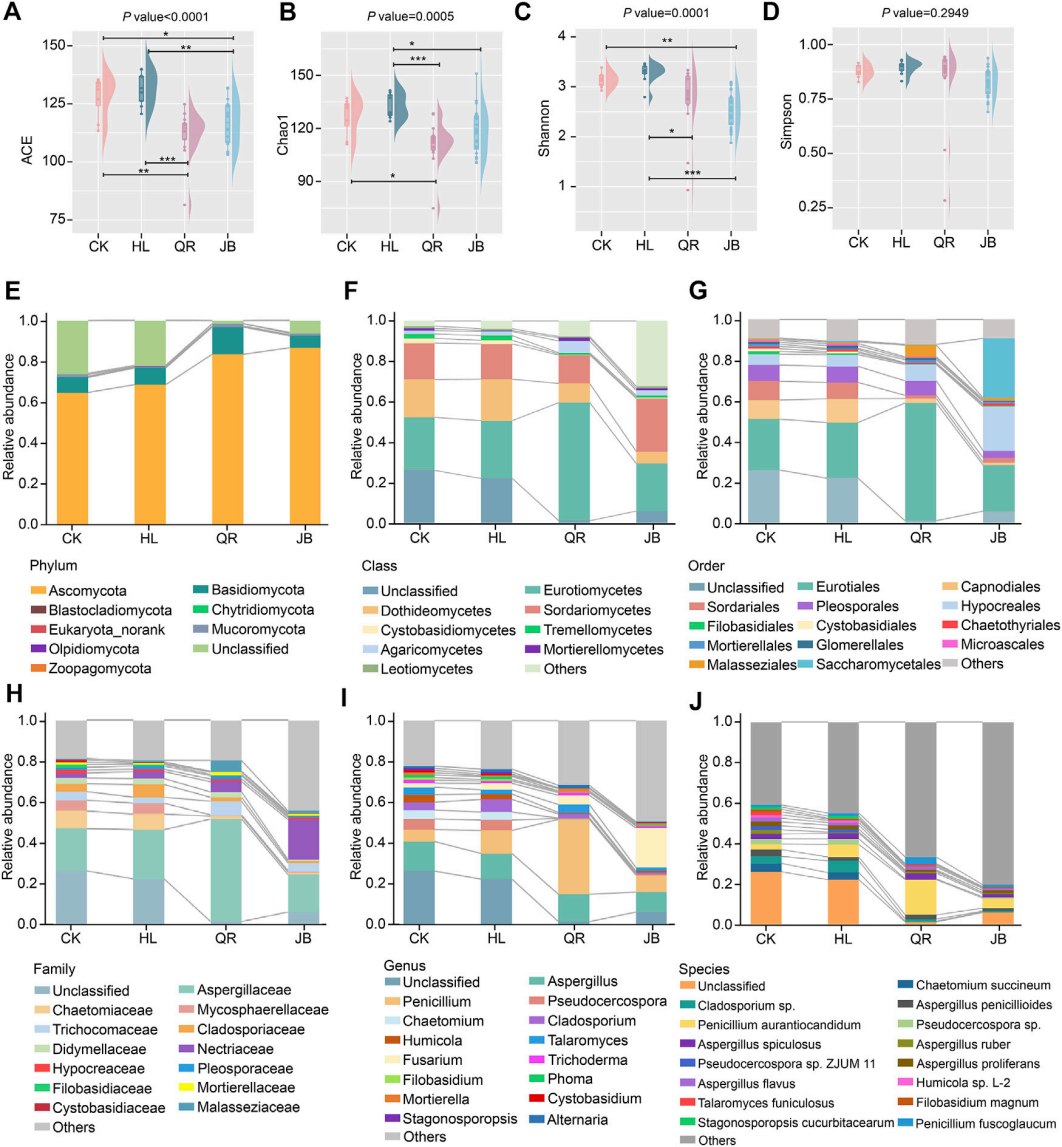
The alteration of gut fungal composition after antipyretic and diaphoretic drugs treatment. The alpha diversity of gut fungi among four groups assessed by **(A)** ACE **(B)** chao1, **(C)** shannon and **(D)** simpson indices. The gut fungal profile diagram at **(E)** phylum **(F)** class, **(G)** order **(H)** family, **(I)** genus and **(J)** species level, respectively. *p < 0.05, **p < 0.01, ***p < 0.001.

The terminology of gut microbiome encloses the whole gut microorganisms, not only bacteria, but also fungi. It has been proved that the balanced gut ecosystem is closely linked to human health. Chao1, ACE, Shannon, and Simpson indices revealed that *Coptis chinensis* had little effect on gut fungal diversity (Figures 4A–D). After combination with antipyretic or diaphoretic drugs, both richness and evenness of gut fungi were notably reduced (Figures 4A–D), suggesting that co-treatment with heat-clearing and exterior-releasing TCMs could enhance the antifungal effect of *Coptis chinensis*, and may even selectively kill specific fungi.

The profile diagram from phylum to species could reflect the specific category difference of different groups. Ascomycota and Basidiomycota are the dominant phyla (Figure 4E), and Eurotiomycetes, Dothideomycetes, and Sordariomycetes represent the dominant classes (Figure 4F). Eurotiomycetes was more abundance in the antipyretic (QR) supergroup, while Dothideomycetes declined in both antipyretic (QR) and diaphoretic (JB) supergroups, especially in the JB supergroup (Figure 4F). At the order level, Malasseziales and Eurotiales were mainly enriched in the antipyretic (QR) supergroup, while Saccharomycetales and Hypocreales were more abundant in the diaphoretic (JB) supergroup. Furthermore, Capnodiales and Sordariales were decreased in both the antipyretic (QR) and diaphoretic (JB) groups (Figure 4G). Notably, the *Penicillium* genera from the Aspergillaceae family was enriched in the antipyretic (QR) supergroup, while the *Fusarium* genera from the Nectriaceae family was enriched in the diaphoretic (JB) supergroup. Moreover, Chaetomiaceae, Mycosphaerellaceae, and Cladosporiaceae families, as well as *Chaetomium* and *Cladosporium* genera were decreased when co-treated with both antipyretic and diaphoretic TCMs (Figures 4H,I). At the species level, co-treatment obviously reduced gut fungal composition as compared to CK and HL groups, among which *Penicillium aurantiocandidum* and *Penicillium fuscoglaucum* were mainly enriched in the antipyretic (QR) supergroup (Figure 4J). These data depicted that antipyretic and diaphoretic TCMs differentially altered *Coptis chinensis*-conditioned gut fungal composition.

### Heat-clearing and exterior-releasing TCMs differentially alters the gut fungal structure

**FIGURE 5:**
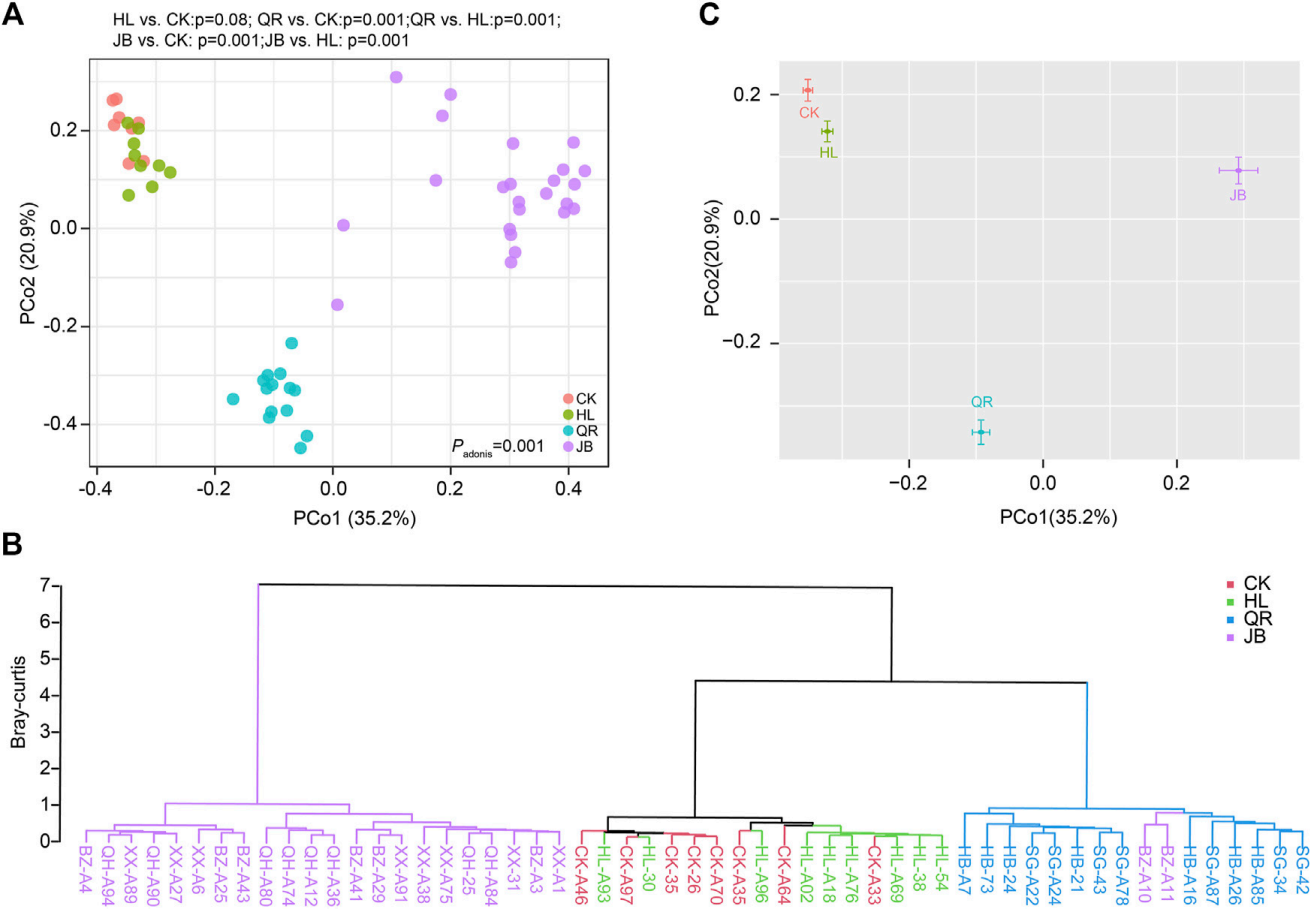
The alteration of gut fungal structure after antipyretic and diaphoretic drugs treatment. The structure of gut fungal was assessed by PCoA analysis which was presented by samples **(A)** or merged groups **(C)**. **(B)** Clustering analysis based on bray-curtis distance.

We also assessed the change in gut fungal structure at the OTU level. As PCoA diagram depicted, *Coptis chinensis* (HL) treatment exerted little effect on gut fungal structure. However, the combination of *Coptis chinensis* with antipyretic and diaphoretic drugs significantly shifted the gut fungal structures from the CK group (Figure 5A). Clustering analysis revealed that the HL group was close to the CK group, while co-treatment with two heat-clearing drugs (HL + HB, HL + SG) obviously altered the gut fungal structure although they were still close to HL group. In contrast, the combination of *Coptis chinensis* with three exterior-releasing TCMs (HL + XX, HL + BZ, and HL + QH) remarkably changed the structure of gut fungi and clustered in another clade (Figure 5B). Together, antipyretic and diaphoretic drugs exerted distinct effects on *Coptis chinensis*-conditioned gut fungi (Figure 5C).

### CAG is a functional gut fungal set responding to heat-clearing and exterior-releasing drugs

**FIGURE 6:**
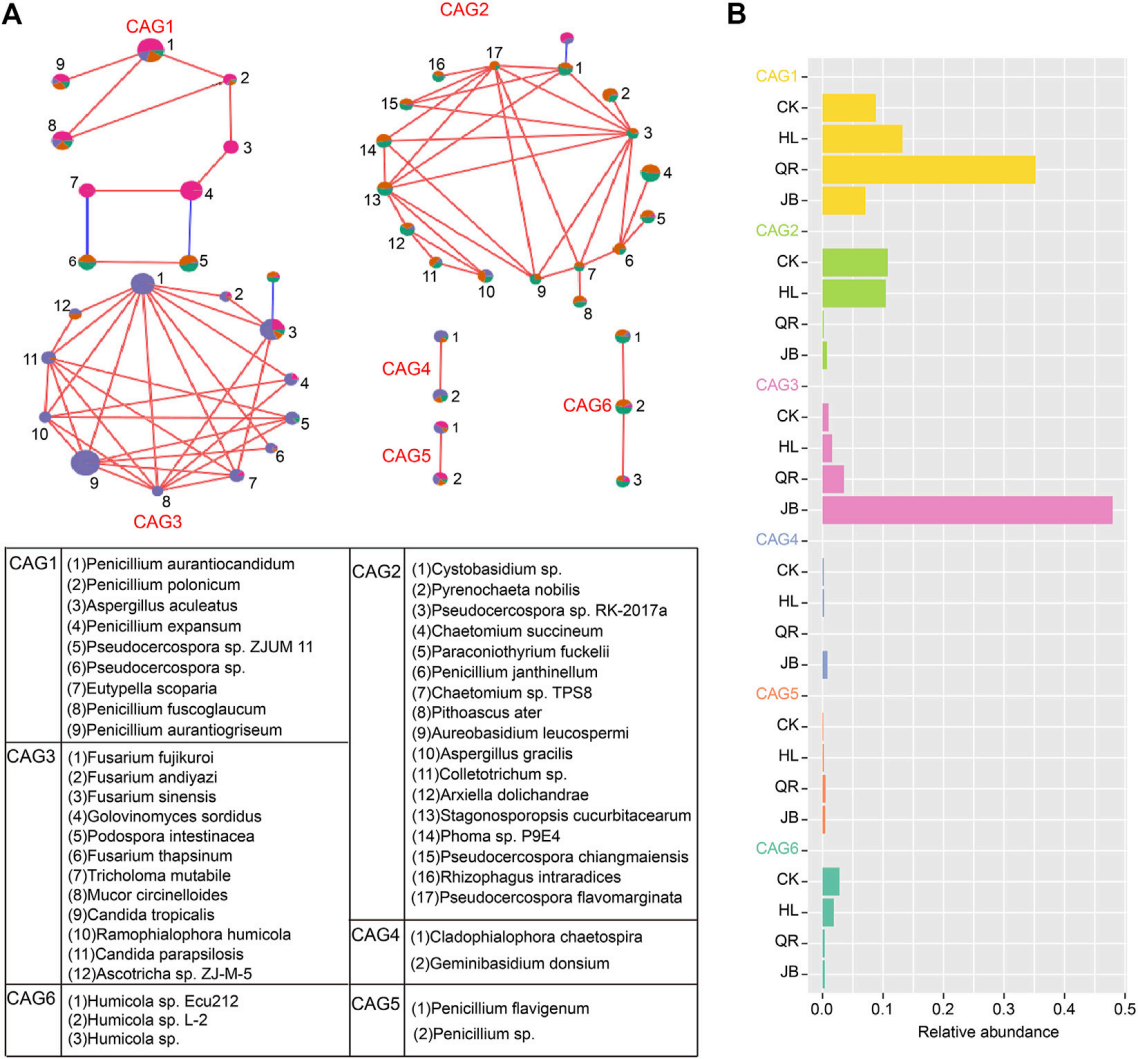
Co-abundance genes group (CAG) as a gut fungal functional set was identified by SparCC analysis **(A)** The composition of five gut fungal CAGs. Red lines represent positive correlation and blue lines represent negative correlation. **(B)** The relative abundance of five CAGs in four groups.

The co-abundance genes group (CAG) is proposed to overcome the challenge of metagenomics analysis from a functional perspective. We therefore conducted SparCC analysis to identify gut fungal CAG. A total of six CAGs were identified in the present study (Figure 6A). CAG1 was dominated by *Penicillium*; CAG2 consists of 17 fungi; CAG3 was composed of *Fusarium* and other fungi. CAG4 contained two species, *Cladophialophora chaetospira*, and *Geminibasidium donsiumin*. CAG5 and CAG6 were composed of *Penicillium* and *Humicola sp.*, respectively. Among these, CAG1 was notably enriched in the heat-clearing TCM supergroup, while CAG3 was significantly abundant in the exterior-releasing supergroup (Figure 6B). These results were consistent with the above analysis at the taxonomy level, that was, *Coptis chinensis* combined with antipyretic medicines enriched *Penicillium*, while combined with diaphoretic medicines enriched *Fusarium*.

### The correlation between gut fungal and bacterial communities

**FIGURE 7:**
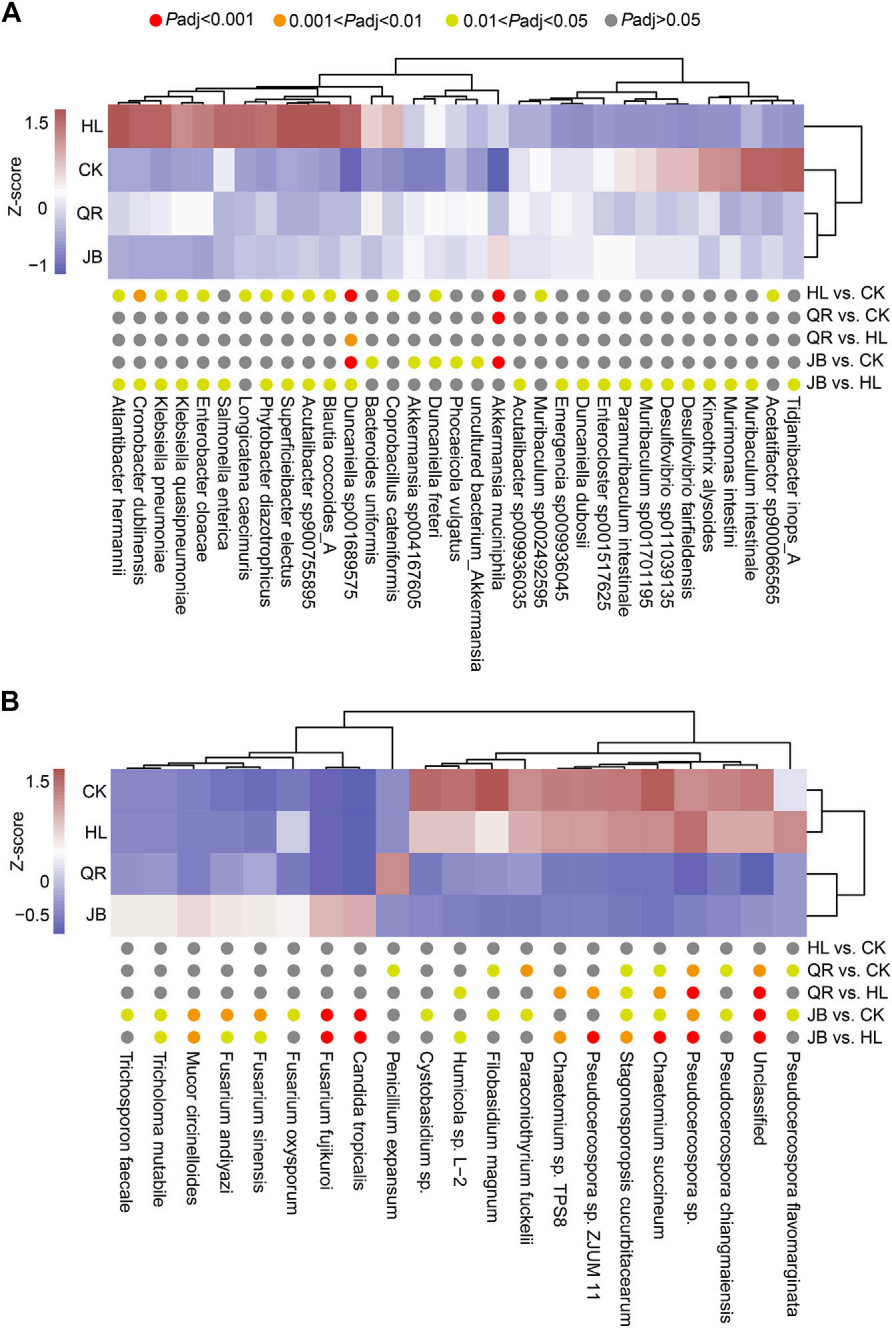
The differential species of gut bacteria and gut fungi. The heat map showed the differential species of **(A)** gut bacteria and **(B)** gut fungi which displayed by Z-score of relative abundance.

To further explore the interaction between gut fungal and bacterial communities, we performed a correlation analysis based on the Spearman algorithm. We first identified the mostly altered gut species by *Coptis chinensis* and heat-clearing/exterior-releasing TCMs. As shown in Figure 7A, *Coptis chinensis* significantly enriched fourteen bacterial species such as *Akkermansia muciniphia*, *Blautia coccoides*, *Klebsiella pneumoniae*, *Klebsiella quasipneumoniae*, *Enterobacter cloacae,* and *Duncaniella sp001689575*, but decreased only two bacterial species *Muribaculum sp002492595* and *Acetatifactor sp900066565*. Compared with *Coptis chinensis*, co-treatment with diaphoretic TCMs led to significant changes in 22 species including most of the *Coptis chinensis*-altered species, while combination with antipyretic TCMs only significantly altered one species *Duncaniella sp001689575*, suggesting that exterior-releasing TCMs are synergistic with *Coptis chinensis*, but heat-clearing TCMs are not. The enrichment of *Akkermansia muciniphia* by *Coptis chinensis* was not deteriorated by either heat-clearing or exterior-releasing TCMs. Analysis of gut fungi revealed that treatment with *Coptis chinensis* did not significantly change any fungi as compared with the control (Figure 7B). Co-treatment with diaphoretic TCMs enriched *Fusarium* spp. (*F. fujikuroi*, *F. oxysporum*, *F. sinensis*, *F. andiyazi*), *Trichosporon faecale*, *Tricholoma mutabile*, and *Mucor circinelloides*, while antipyretic medicines only enriched *Penicillium expansum* (Figure 7B).

**FIGURE 8:**
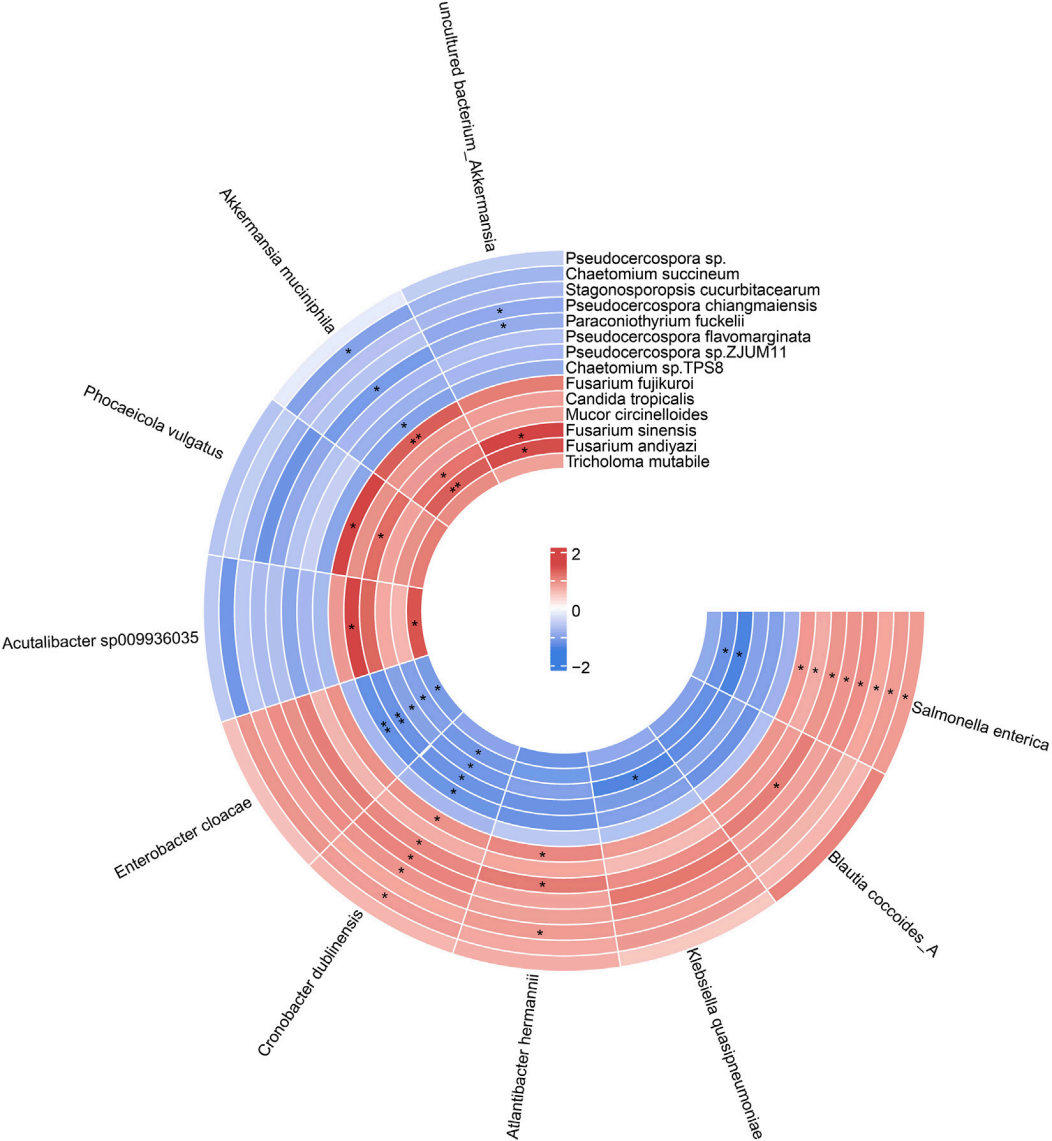
The circular heat map showed the correlation between differential species of gut fungi and gut bacteria. *p* value was adjusted by FDR. *Padj < 0.05, ** *P*adj < 0.01.

We next performed correlation analysis based on the Spearman algorithm to explore the relationship between gut fungal and bacterial communities. We found *Akkermansia muciniphila* was positively associated with *Fusarium fujikuroi*, *Fusarium sinensis*, and *Fusarium andiyazi*, while negatively correlated with *Chaetomium succineum*, *Paraconiothyrium fuckelii*, and *Chaetomium sp. TPS8*. In addition, the *Coptis chinensis* enriched species, such as *Blautia coccoides*, *Klebsiella quasipneumoniae*, and *Enterobacter cloacae*, were positively correlated with *Pseudocercospora flavomarginata*, while negatively related to *Fusarium* (Figure 8). These results suggested that there are complex interactions between gut fungal and bacterial communities. The differential modulation of heat-clearing and exterior-releasing TCMs on gut fungi may be responsible for their distinct pharmacological effects.

### Chemical components of the heat-clearing and exterior-releasing TCM pairs

**FIGURE 9:**
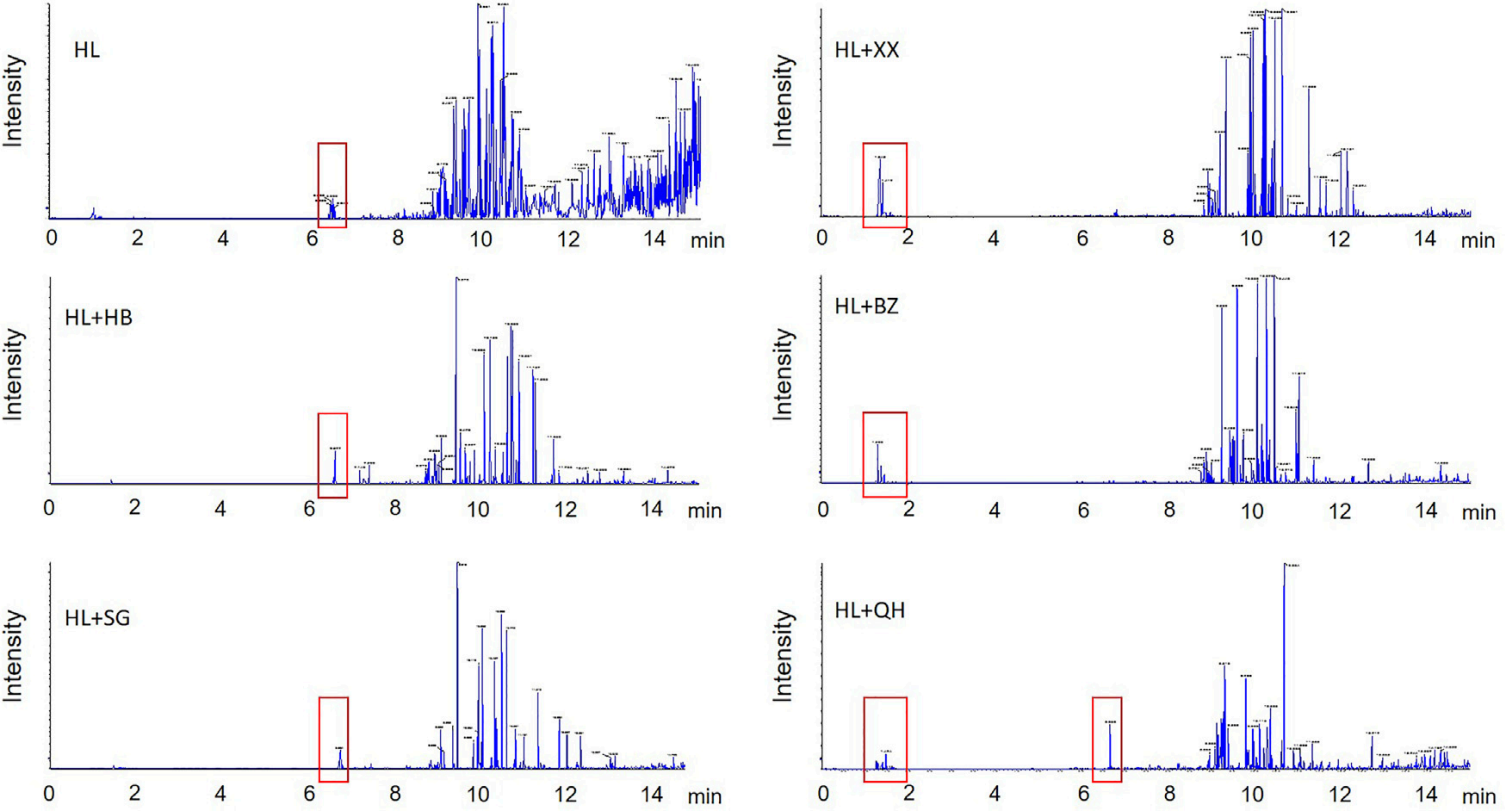
The secondary ion mass spectrogram of the heat-clearing and exterior-releasing TCM pairs by UPLC-QTOF/MS analysis. HL, *Coptis chinensis*; HB, *Phellodendron chinense*, SG, Gypsum Fibrosum, XX, *Asari radix et rhizoma*, BZ, *Angelicae dahuricae radix*, QH, *Notopterygii rhizoma et radix*.

The six antipyretic (heat-clearing) and diaphoretic (exterior-releasing) TCMs are popular medical herbs in traditional Chinese medicine practice. The chemical components in their aqueous extracts have been well-investigated (Table 1). The main components of the two heat-clearing TCMs, *Coptis chinensis Franch* (HL) and *Phellodendron chinense C.K.Schneid*. (HB) were alkaloids, with berberine being the most abundant compound (Zhao et al., 2020). The other heat-clearing TCM, *Gypsum Fibrosum* (Shigao, SG) is mainly composed of CaSO_4_.2H_2_O. The three exterior-releasing TCMs comprise high content of essential oils as main components (Li et al., 2015; Zhang et al., 2016). We analyzed the chemical constituents of the TCM pair extracts by UPLC-QTOF/MS (Figure 9). Overall, the chemical profiles of the six tested extracts in the secondary mass spectrogram had a strong resemblance, with the exterior-releasing TCM pairs enriched components with retention time at 1–2 min while the heat-clearing TCM pairs and *Coptis chinensis Franch-Notopterygii rhizoma et radix* (HL + QH) pair enriched chemicals with retention time around 6.5 min.

**TABLE 1:** Main chemical components of the tested heat-clearing and exterior-releasing TCMs.

TCM	Activity	Main chemical compoents
*Coptis chinensis* Franch (HL)	Heat-clearing	Alkaloids (berberine, coptisine, worenine, palmatisine, jatrorrhizne, epiberberine, groenlandicine, berberastine)
*Phellodendron chinense* C.K.Schneid. (HB)	Heat-clearing	Alkaloids (berberine, jatrorrhizine, magnoliazine, phellodendrine, palmatine, dauricine)
*Gypsum Fibrosum* (SG)	Heat-clearing	CaSO_4_.2H_2_O
*Asarum sieboldii* Miq (XX)	Exterior-releasing	Essential oils (methyl eugenol, safrole, eucarvone, elemene, asarone, acaryl ether, cetene, cineole), lignans (L-asarone, L-sesamin and kakul alcohol) and flavonoids
*Angelicae dahuricae radix* (BZ)	Exterior-releasing	Essential oils (octadecanol, cyclododecane); Coumarins (imperatorin, isoimperatorin, oxypeucedanin)
*Notopterygii rhizoma et radix* (QH)	Exterior-releasing	Essential oils (α-thujene, α、β-pinene, β-ocimene, γ-terpinene, limonene, 4-terpinenol, bornylacetate), α-copaene, β-farnesene, guaiol); Coumarins (isoimperatorin, cnidilin,notopterol, bergapten, demethylfuroinnarin)

The clustering analysis of the heat-clearing and exterior-releasing TCM pairs on gut fungal data revealed that the heat-clearing TCMs and exterior-releasing TCMs clustered together and separated from each other (Figure 5B), implying that the chemicals with retention time at 1–2 min might account for the fungi modulation by these TCMs. In contrast, although *Notopterygii rhizoma et radix* (QH) is an exterior-releasing TCM, the group treated with HL + QH pair was closer to that treated with heat-clearing Gypsum Fibrosum (Figure 3B), suggesting that the components with retention time around 6.5 min might participate in the regulation of gut bacteria. These specific compounds may be of importance to distinguish the differential effect of heat-clearing and exterior-releasing TCMs on gut bacteria and fungi and will be analyzed in the future.

## Discussion

Antipyretic (heat-clearing) drugs and diaphoretic (exterior-releasing) drugs are the top two types of TCMs that are commonly used in the clinic. They both possess anti-microbial effects, but their anti-microbial spectrums remain largely unknown, nor do we know whether they are synergistic in the inhibition of gut pathogens. In this work, we found *Coptis chinensis* significantly changed the structure of gut bacteria but showed little influence on gut fungi. Combination with antipyretic or diaphoretic TCMs alleviated the impact of *Coptis chinensis* on gut bacteria to a similar degree. However, both heat-clearing and exterior-releasing TCMs significantly strengthened the influence of *Coptis chinensis* on gut fungi, with the exterior-releasing TCMs superior to the heat-clearing drugs. Two kinds of fungi, *Penicillium* spp. and *Fusarium* spp., were especially enriched by antipyretic and diaphoretic TCMs, respectively. Furthermore, the diaphoretic TCMs-enriched *Fusarium* spp. were positively related to gut bacteria *Akkermansia* spp. which can interact with Toll-like receptor 4 (TLR4) and regulates thermogenesis, thus providing a potential linkage with their pro-diaphoresis effect. To our best knowledge, this is the first study devoted to investigating the differential effects of traditional heat-clearing and exterior-releasing TCMs on both gut bacteria and gut fungi.

*Coptis chinensis*, a representative heat-clearing TCM with cold properties, is widely applied to treat fever-related diseases including inflammation, cancer, diarrhea, etc (Danielewski et al., 2022; Gao and Lv, 2022). In clinical practice, *Coptis chinensis* is used alone or in formula to attenuate the symptoms of fever, bloated and painful abdomen, as well as dry stools caused by “internal heat" (Tseng et al., 2006). A large number of pharmacological investigations have proved that gut microbiota is an essential target for medicating the molecular mechanism of *Coptis chinensis*. *Coptis chinensis* and its active compound berberine markedly enriched beneficial gut bacteria, such as *Akkermansia*, *Blautia*, *Bacteroides*, *Butyricoccus*, and *Phascolarctobacterium* (Zhang et al., 2015; Dong et al., 2021; Wu C. et al., 2022). However, the influence of *Coptis chinensis* on gut fungi is scarcely investigated, although an *in vitro* test showed *Coptis chinensis* could inhibit *Candida* (Zhang et al., 2019). In our study, we confirmed the promoting effect of *Coptis chinensis* on *Akkermansia muciniphila*, *Blautia coccoides*, *Bacteroides uniformis*, and *Bacteroides stercorirosoris*. Furthermore, we also systemically evaluated the impact of *Coptis chinensis* on the gut fungal community. In contrast with gut bacteria, oral administration of *Coptis chinensis* showed little effect on gut fungi from both diversity and overall structure aspects. Detailed analysis of individual fungal taxa revealed that *Coptis chinensis* increased the abundance of *Penicillium* and *Fusarium* and decreased *Filobasidium* and *Mortierella*. *Penicillium* is a well-known fungus producing multiple antibiotics including penicillin (Samuel et al., 2022), whose enrichment may contribute to *Coptis chinensis*’s anti-microorganism effect. Although most *Coptis chinensis*-altered fungi are functionally unknown. Our results provide a first clue for the potential modulation of *Coptis chinensis* on gut fungal community.

Antipyretic drugs and diaphoretic drugs are the most commonly used two classes of TCMs in clinics to treat fever-related disease, such as *Phellodendron amurense*, Angelicae dahuricae radix, and Notopterygii hizome et radix. It has been demonstrated that the diaphoretic drug Notopterygii hizome et radix could regulate Prevotellaceae*_NK3B319*_group, *Ruminococcus*, *Clostridium_sensu_stricto*_*1*, *Blautia*, and *Anaerostipes* (Wu S.-S. et al., 2022), while the antipyretic drug *Phellodendron amurense* could increase *Roseburia*, *Clostridium*, *Odoribacter*, and *Parabacteroides*, as well as specifically enriched *Oscillospira* and *Rikenella* (Xu et al., 2020). In clinical practice, TCMs are usually combined with each other to elevate efficacy or reduce side effects. However, the interactions between different TCM drugs on gut microbiota have not been fully investigated. Our results showed that the antipyretic drugs combined with *Coptis chinensis* could restore the perturbated gut microbiota by *Coptis chinensis* to different degrees. Among which, Gypsum Fibrosum (Shigao, SG) was able to migrate the microbial structure toward normal, while *Phellodendron chinense* (Huangbo, HB) had little effect. Comparatively, all three diaphoretic drugs combined with *Coptis chinensis* essentially restored the gut microbial homeostasis, indicating that combination use of different types of drugs could effectively alleviate toxic and side effects. Simultaneously, the combined use of antipyretic drugs and diaphoretic drugs also enhanced the beneficial effect of *Coptis chinensis* on gut microbiota. For example, the *Coptis chinensis*-enriched *Akkermansia muciniphila* was further enhanced, while opportunistic pathogens *Enterobacter cloacae*, *Klebsiella pneumoniae* and *Klebsiella quasipneumoniae* were reduced when co-administration of diaphoretic drugs as compared with *Coptis chinensis* alone. These results proved that the combination of different drugs can indeed elevate their efficiency and reduce side effects.

In the present study, we reported the modulation effects of *Coptis chinensis* and its herbal pairs on gut fungi for the first time. *Coptis chinensis* had little effect on the gut fungi community, but when combined with antipyretic and diaphoretic TCMs, both gut fungal structure and composition were notably changed, showing a strong synergistic effect. Antipyretic- and diaphoretic-associated fungal alteration was characterized by decreased biodiversity and significantly altered structure. Specifically, *Fusarium* (*F. fujikuroi*, *F. sinensis* and *F. andiyazi*) and *Tricholoma mutabile* were notably increased in the diaphoretic (JB) supergroup, while *Chaetomium succineum* and *Pseudocercospora sp. ZJUM 11* were decreased. Moreover, *Pseudocercospora sp*., *Pseudocercospora sp. ZJUM 11*, *Pseudocercospora chiangmaiensis*, *Chaetomium succineum*, *Chaetomium sp. TPS8* and *Stagonosporopsis cucurbitacearum* were reduced after antipyretic TCMs treatment, while *Penicillium polonicum* and *Penicillium aurantiogriseum* were obviously enriched in the antipyretic (QR) supergroup. Another key finding of the current study is that the modulation effect of diaphoretic drugs combined with *Coptis chinensis* on gut microbiota was more obvious than antipyretic drugs, suggesting that diaphoretic drugs combined with *Coptis chinensis* had a unique regulatory ability for gut fungi. These results revealed that gut fungi may be an important indicator to distinguish antipyretic and diaphoretic drugs.

Although the alteration of the gut fungal community was obvious after antipyretic and diaphoretic herb pairs treatment, the physiological significance was vague due to the lacking of related functional studies. It is well known that the gut micro-environment is an intricate system with a complex interaction between gut bacteria and gut fungi. Hence, investigating the relationship between bacteria and fungi contributes to elucidating the alteration of gut fungi and the mechanism of antipyretic and diaphoretic drugs. In our study, though we found *Penicillium* was enriched in the antipyretic drug treatment group, a significant interaction was not presented between *Penicillium* and gut bacteria, indicating that antipyretic drugs possibly tend to affect gut fungal communities than fungi-bacteria interaction. However, we found that *Fusarium* shared a positive co-occurring correlation with *Akkermansia muciniphila*, and a negative relationship with *Enterobacter cloacae*, *Salmonella enterica*, and *Cronobacter dublinensis*. *Akkermansia muciniphila* is a known beneficial bacterium that could elevate the concentration of butyrate. Spearman correlation analysis also showed that the abundance of *Fusarium* was positively associated with isobutyrate (Li et al., 2020). A recent study reported that *Akkermansia* can interact with Toll-like receptor 4 (TLR4) (Liu et al., 2022) which further regulates thermogenesis (Muneoka et al., 2019). Therefore, the positive relation between *Fusarium* and *Akkermansia* may link the antithermic effect of *Fusarium*-enriching diaphoretic drugs. Our results therefore provide the first gut-related evidence for the sweating and heat-clearing effect of diaphoretic drugs. The interaction between gut bacteria and gut fungi will contribute to interpreting the *in vivo* characteristics and pharmacological mechanisms of diaphoretic drugs and antipyretic drugs.

In light of our finding that gut fungi differentially respond to the impact of heat-clearing and exterior-releasing TCMs on *Coptis chinensis*-conditioned gut microbiota, we have to acknowledge a handful of limitations on the present study. For example, we did not build the animal fever model to assess the pharmacological efficacy of antipyretic and diaphoretic drugs, did not detect the indicators of their potential effects, and did not link the relationship between these indicators and the alterations in the gut microbiome, especially gut fungal species. For the reliability of these study outcomes and for the explanation of the functional characteristics of those two kinds of TCMs, we will design a more rigorous and logical experiment in the near future to explore how gut fungi and gut bacteria participate in regulating the efficacy of TCMs. Moreover, some new methods will be applied to investigate the relationship between alterations of gut microbiome caused by different TCMs and clinical effect features, as well as identify the active component from herbs directly acting on the gut microbiome, so as help to further understand the important role of the property of TCM (Wang et al., 2018; Chen et al., 2019; Huo et al., 2022; Peng et al., 2022).

## Conclusion

In conclusion, our study focused on the modulation of both gut fungi and gut bacteria and revealed that *Coptis chinensis* combined with diaphoretic TCMs could enrich bacterial *Akkermansia* and fungal *Fusarium*, and possibly reshaped the correlation between gut fungi and gut bacteria to exert its efficacy. While *Coptis chinensis* combined with antipyretic TCMs could enrich *Penicillium* and then influence its efficacy.

## Data Availability

The original contributions presented in the study are included in the article/supplementary material, further inquiries can be directed to the corresponding author.

## Abbreviations

TCM, traditional Chinese medicine; JB, exterior-releasing; QR, heat-clearing; HL, Coptis chinensis Franch; HB, Phellodendron chinense C.K.Schneid; SG, Gypsum Fibrosum; XX, Asarum sieboldii Miq; BZ, Angelica dahurica (Hoffm) Benth. & Hook.f. ex Franch. & Sav; QH, Notopterygium incisum KC.Ting ex HT. Chang; PCoA, principal coordinate analysis; ITS1/2, internal transcribed spacer 1/2; CAGs, co-abundance genes groups; SCFAs, short-chain fatty acids; TLR4, Toll-like receptor 4.
